# P. Preterre

**Published:** 1871-07

**Authors:** 


P. Preterre, D.D.S., a graduate of the Pennsylvania College of Dental Surgery, died in the city of New York on October 30th, 1870, aged 58 years. Dr. Preterre was the oldest of five brothers, all practicing dentists.

